# Pre-existing interstitial lung disease is associated with onset of nivolumab-induced pneumonitis in patients with solid tumors: a retrospective analysis

**DOI:** 10.1186/s12885-021-08661-3

**Published:** 2021-08-16

**Authors:** Teppei Yamaguchi, Junichi Shimizu, Takaaki Hasegawa, Yoshitsugu Horio, Yoshitaka Inaba, Nobuhiro Hanai, Kei Muro, Toyoaki Hida

**Affiliations:** 1 Department of Thoracic Oncology, Aichi Cancer Center Hospital, 1-1, Kanokoden, Chikusa-ku, Nagoya, Aichi 464-8681 Japan; 2 Department of Diagnostic and Interventional Radiology, Aichi Cancer Center Hospital, 1-1, Kanokoden, Chikusa-ku, Nagoya, Aichi 464-8681 Japan; 3 Department of Head and Neck Surgery, Aichi Cancer Center Hospital, 1-1, Kanokoden, Chikusa-ku, Nagoya, Aichi 464-8681 Japan; 4 Department of Clinical Oncology, Aichi Cancer Center Hospital, 1-1, Kanokoden, Chikusa-ku, Nagoya, Aichi 464-8681 Japan

**Keywords:** Non-small cell lung cancer, Head and neck cancer, Gastric cancer, Pneumonitis, Nivolumab, PD-1

## Abstract

**Background:**

Nivolumab, an anti-programmed death 1 (PD-1) monoclonal antibody, has shown survival benefit in clinical trials of various malignant tumors. Nivolumab-induced pneumonitis is major immune-related adverse event (irAE) that is occasionally serious and life-threatening. The aim of this study was to examine the association between pre-existing interstitial lung disease (ILD) on chest computed tomography (CT) and nivolumab-induced pneumonitis among different types of solid tumors.

**Methods:**

We retrospectively collected the clinical data of 311 patients who were diagnosed with non-small cell lung cancer (NSCLC), head and neck cancer (HNC), or gastric cancer (GC), and treated with nivolumab monotherapy. Patients who underwent chest CT immediately before starting nivolumab without previous thoracic radiotherapy or other immune checkpoint inhibitors were eligible. We collected baseline patient characteristics and assessed pre-existing ILD on baseline chest CT.

**Results:**

Finally, 188 patients were included in the analysis: 96 patients with NSCLC, 43 patients with HNC, and 49 patients with GC. NSCLC patients had a significantly higher rate of pre-existing ILD compared with HNC/GC patients (*P* = 0.047). Nivolumab-induced pneumonitis occurred in 11.7% (22 of 188), including 14.6% (14 of 96) of NSCLC, and 8.7% (8 of 92) of HNC/GC. Univariate and multivariate logistic regression analyses revealed that pre-existing ILD (odds ratio, 5.92; 95% confidence interval (CI), 2.07–18.54, *P* = 0.0008) and male sex (odds ratio, 5.58; 95% CI, 1.01–104.40, *P* = 0.049) significantly increased the risk of nivolumab-induced pneumonitis.

**Conclusion:**

Our results indicated that pre-existing ILD and male sex are risk factors for nivolumab-induced pneumonitis in solid tumors.

**Supplementary Information:**

The online version contains supplementary material available at 10.1186/s12885-021-08661-3.

## Background

Nivolumab is a selective, fully humanized IgG4 monoclonal antibody that blocks binding between programmed death 1 (PD-1) and programmed death ligand-1 (PD-L1)/PD-L2, encouraging the antitumor activity of T cells. Nivolumab has demonstrated antitumor efficacy in clinical trials of various malignant tumors, including melanoma, renal cell cancer, non-small-cell lung cancer (NSCLC), head and neck cancer (HNC), and gastric cancer (GC) [1–6]. However, immune check point inhibitors (ICI) including nivolumab can induce immune-related adverse events (irAEs) such as skin rash, colitis, endocrine disorders, hepatotoxicity, and pneumonitis.

Among them, pneumonitis is a relatively common but potentially life-threatening irAE. We recently reported that pre-existing interstitial lung diseases (ILD) is a risk factor for anti-PD-1-induced pneumonitis in patients with NSCLC; however, it is not clear if this tendency applies to other types of tumors [7]. Although the incidences of most irAEs are considered to be similar regardless of tumor type, in the case of pneumonitis these frequencies may differ across tumor types. In the previous phase III clinical trials of nivolumab, the incidence of nivolumab-induced pneumonitis was reported as 3.5–5% in NSCLC, 2.1% in squamous cell HNC, and < 1% in GC, and thus it appears to vary among the different cancer types [2–5]. We speculated that these tumor-specific bias in the incidence of pneumonitis may be related to pre-existing ILD, regardless of the tumor type. Moreover, patients complicated with ILD are routinely excluded from clinical trials, therefore, these questions need to be resolved by real-world data.

The aim of this study was to evaluate the association between ILD on chest computed tomography (CT) and nivolumab-induced pneumonitis among cohorts with different tumor types, including NSCLC, HNC, and GC.

## Methods

### Patients

We retrospectively reviewed 311 patients who were diagnosed with NSCLC, HNC, or GC and treated with nivolumab monotherapy at Aichi Cancer Center Hospital, Japan between 17 December 2015 and 30 April 2018. Patients who underwent chest CT immediately before the start of nivolumab treatment were included in the analysis. Patients who received other chemotherapy regimens between the last chest CT and nivolumab and who previously received thoracic radiotherapy or any immune checkpoint inhibitors before nivolumab were excluded. All patients were treated with nivolumab monotherapy at 3 mg/kg every 2 weeks. The following data at the time of nivolumab initiation were collected from medical records: age, sex, smoking status, performance status, comorbidities, serum levels of C-reactive protein, serum levels of lactate dehydrogenase, neutrophil-to-lymphocyte ratio, chest CT before nivolumab, number of treatment cycles of nivolumab, and date and severity of nivolumab-induced pneumonitis.

### Radiographic analysis

All patients in our study were examined with a helical CT scanner with a slice thickness of 1–10 mm (mostly 5 mm). Two diagnostic radiologists (T. H. and Y. I.) evaluated baseline chest CT findings according to the American Thoracic Society (ATS)/European Respiratory Society (ERS)/Japanese Respiratory Society (JRS)/Latin American Thoracic Association (ALAT) CT criteria for usual interstitial pneumonia (UIP). Pre-existing ILD was divided into three types, UIP pattern, possible UIP pattern, and inconsistent with UIP pattern [8]. The clinical data were blinded, and cases of discrepancy were resolved by consensus. The diagnosis of nivolumab-induced pneumonitis was confirmed by two pulmonologists (T. Y. and J. S.) according to the National Cancer Institute Common Toxicity Criteria for Adverse Events, version 4.0, with reference to careful examination to exclude other pulmonary diseases such as infection, cancer progression, congestive heart failure, and pulmonary thromboembolism [9].

### Statistical analysis

Differences in characteristics between the two cohorts were evaluated using the Chi-square test or the Fisher’s exact test (if the expected frequency is lower than five) to compare categorical variables and Mann-Whitney *U* test to compare continuous variables. Univariate and multivariate logistic regression analyses were conducted to assess the potential independent risk factors for pneumonitis associated with nivolumab. All variables with *P*-values < 0.1 in the univariate analysis were entered into the multivariate analyses. All tests were two-sided with a significance level of *P* < 0.05. All analyses were preformed using JMP version 11.0 statistical software (SAS Institute Inc., Cary, NC, USA). The analysis cut-off date was 31 July 2018.

## Results

### Patient characteristics

**Fig. 1:**
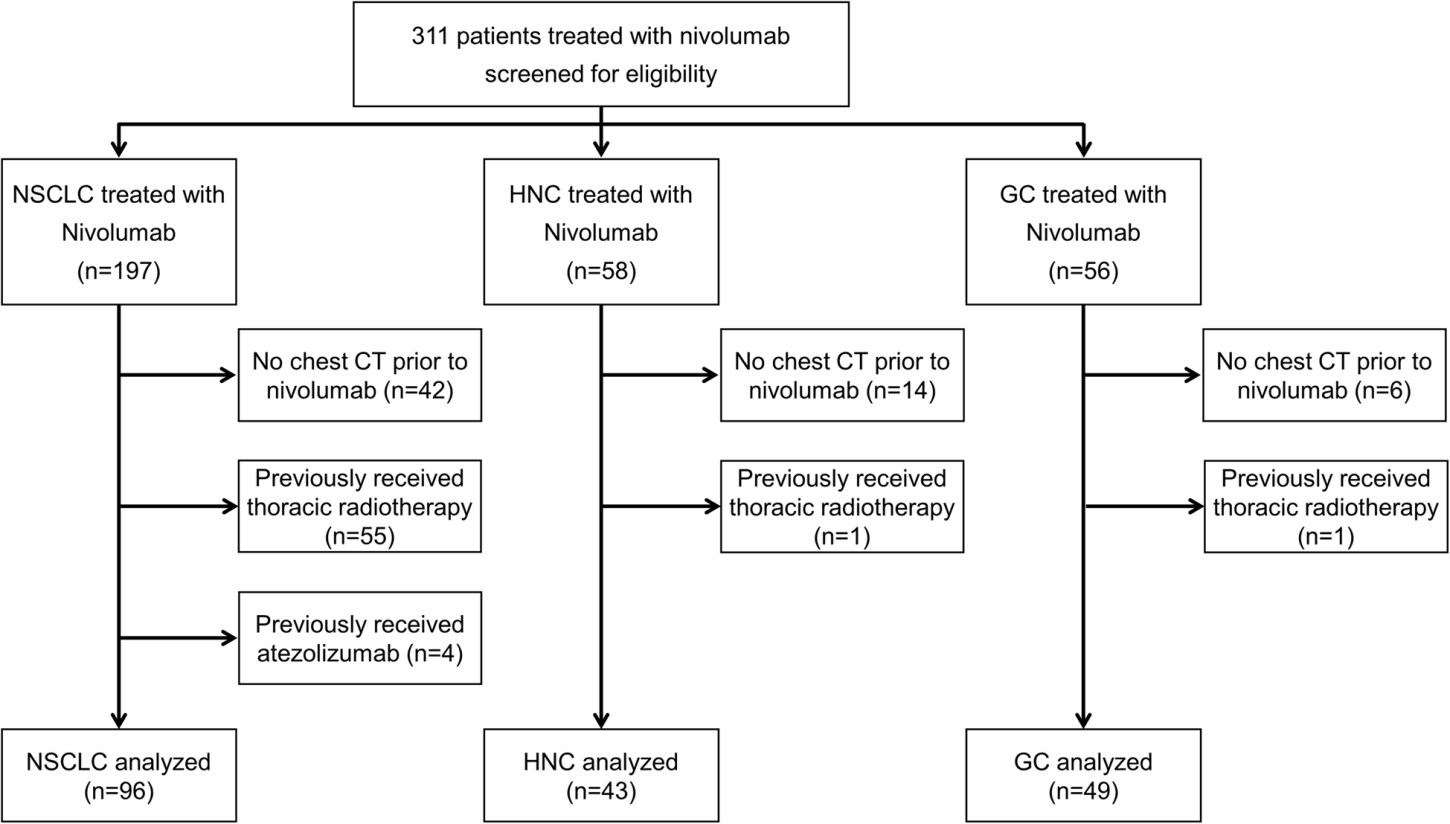
Patient flowchart. NSCLC, non-small cell lung cancer; HNC, head and neck cancer; GC, gastric cancer; CT, computed tomography

We screened 311 consecutive patients with solid tumors who received nivolumab and excluded 123 patients: 62 patients with no chest CT prior to nivolumab, 57 patients who previously received thoracic RT, and four patients who previously received atezolizumab. Finally, 188 patients were analyzed in our study, including 96 patients with NSCLC, 43 patients with HNC, and 49 patients with GC (Fig. 1). Patient characteristics of the NSCLC group and the HNC/GC group are summarized in Table 1 and Supplemental Table S1. No significant differences were detected in age, sex, smoking status, performance status, and number of treatment cycles of nivolumab between the NSCLC group and the HNC/GC group. For comorbidities, the incidence of chronic obstructive pulmonary disease (COPD) was significantly higher in the NSCLC group. In the analysis of pre-existing ILD on chest CT, the NSCLC group had a significantly higher rate of pre-existing ILD compared to the HNC / GC group (*P* = 0.047). The majority of patients were classified as “non-fibrosis”, however, 26 patients (27.1%) in the NSCLC group and 14 patients (15.2%) in the HNC/GC group had pre-existing ILD on chest CT. Based on the ATS/ERS/JRS/ALAT CT criteria for UIP, 17 patients (17.7%) were classified as inconsistent with UIP, three (3.1%) as possible UIP, and six (6.3%) as UIP in the NSCLC group, while in the HNC/GC group, 11 (12.0%) patients were classified as inconsistent with UIP, one (1.1%) as possible UIP, and two (2.2%) as UIP. In addition, pre-existing ILD was predominant in male patients, accounting for 38 of 133 patients (28.6%). While in females, only two of 55 patients (3.6%) had pre-existing ILD and were classified as “inconsistent with UIP.”

**Table 1:** Patient characteristics

Characteristics	NSCLC *n* = 96 n, (%)	HNC/GC *n* = 92 n, (%)	*P*-value
Age, years			
Median (range)	68 (44–80)	67 (24–85)	0.46
< 65	33 (34.4)	38 (41.3)	
≥ 65	63 (65.6)	54 (58.7)	
Sex			0.98
Male	68 (70.8)	65 (70.7)	
Female	28 (29.2)	27 (29.3)	
Smoking status			0.40
Current/ex-smoker	75 (78.1)	67 (72.8)	
Current smoker	5 (5.2)	11 (12.0)	
Ex-smoker	70 (72.9)	56 (60.9)	
< 20 pack-years	12 (12.5)	18 (19.6)	
≥ 20 pack-years	63 (65.6)	45 (48.9)	
Unknown pack-years	0	4 (4.3)	
Never-smoker	21 (21.9)	25 (27.2)	
Performance status			0.36
0–1	82 (85.4)	74 (80.4)	
≥ 2	14 (14.6)	18 (19.6)	
Prevalence of patient comorbidities			
Arterial hypertension	30 (31.3)	26 (28.3)	0.65
Cardiovascular disease	5 (5.2)	8 (8.7)	0.35
Diabetes	11 (11.5)	12 (13.0)	0.74
COPD	12 (12.5)	3 (3.3)	0.029^a^
No. of treatment cycles of nivolumab			
Median (range)	5 (1–58)	5 (1–25)	0.42
Pre-existing ILD on chest CT			
Normal	70 (72.9)	78 (84.8)	0.047
Pre-existing ILD	26 (27.1)	14 (15.2)	
UIP	6 (6.3)	2 (2.2)	
Possible UIP	3 (3.1)	1 (1.1)	
Inconsistent with UIP	17 (17.7)	11 (12.0)	

*NSCLC* non-small cell lung cancer, *HNC* head and neck cancer, *GC* gastric cancer, *COPD* chronic obstructive pulmonary disease, *ILD* interstitial lung disease, *CT* computed tomography, *UIP* usual interstitial pneumonia, ^a^ Fisher’s exact test

### Incidence of nivolumab-induced pneumonitis and UIP diagnosis categories

Incidence of nivolumab-induced pneumonitis according to UIP diagnosis categories are listed in Table 2. The overall nivolumab-induced pneumonitis occurrence rate was 11.7% (22 of 188) and the grade 3 or higher pneumonitis rate was 1.6% (3 of 188). In an analysis based on tumor type, nivolumab-induced pneumonitis occurred in 14.6% (14 of 96) of the NSCLC group (grade 1, seven patients; grade 2, five patients; and grade 3, two patients), and in 8.7% (eight of 92) of the HNC/GC group (grade 1, five patients; grade 2, two patients; and grade 5, one patient). Eight of grade 1 patients were underwent follow-up, and the remaining 14 patients received steroids. Of the 22 patients with pneumonitis, one developed pneumonitis after discontinuing treatment with another irAE (cholangitis), 18 patients discontinued treatment, and three patients continued to receive nivolumab without exacerbation of pneumonitis. Four of the 18 patients who discontinued nivolumab resumed nivolumab but did not experience relapse of pneumonitis. The median time to onset of pneumonitis from starting nivolumab was 63 days (range, 6–634 days) in the NSCLC group and 56 days (range, 2–107 days) in the HNC/GC group. In cases with no fibrosis, pneumonitis occurred in 5.4% (eight of 148 patients), including 5.7% (four of 70) of NSCLC and 5.1% (four of 78) of HNC/GC. Whereas in cases with pre-existing ILD, pneumonitis complicated 35.0% (14 of 40 patients), including 38.5% (10 of 26 patients) of NSCLC and 28.6% (four of 14 patients) of HNC/GC. According to an analysis of the ATS/ERS/JRS/ALAT CT criteria for UIP, pneumonitis was observed in 32.1% (nine of 28) patients with “inconsistent with UIP” and in 41.7% (five of 12) with “possible UIP/UIP.” Grade 3 or higher pneumonitis developed only in cases with pre-existing ILD, two cases of grade 3 were in NSCLC with inconsistent with UIP and UIP, and another case of grade 5 was HNC with possible UIP.

**Table 2:** Incidence of nivolumab-induced pneumonitis and UIP diagnosis categories

	Total, n	Any grade, n (%)	≥Grade 3, n (%)
Total	188	22 (11.7)	3 (1.6)
NSCLC	96	14 (14.6)	2 (2.1)
HNC / GC	92	8 (8.7)	1 (1.1)
No fibrosis, all tumor types	148	8 (5.4)	0
No fibrosis, NSCLC	70	4 (5.7)	0
No fibrosis, HNC/GC	78	4 (5.1)	0
Pre-existing ILD, all tumor types	40	14 (35.0)	3 (7.5)
Inconsistent with UIP, all tumor types	28	9 (32.1)	1 (3.6)
Possible UIP/UIP, all tumor types	12	5 (41.7)	2 (16.7)
Pre-existing ILD, NSCLC	26	10 (38.5)	2 (7.7)
Inconsistent with UIP, NSCLC	17	6 (35.3)	1 (5.9)
Possible UIP/UIP, NSCLC	9	4 (44.4)	1 (11.1)
Pre-existing ILD, HNC/GC	14	4 (28.6)	1 (7.1)
Inconsistent with UIP, HNC/GC	11	3 (27.3)	0
Possible UIP / UIP, HNC/GC	3	1 (33.3)	1 (33.3) ^a^

*NSCLC* non-small cell lung cancer, *HNC* head and neck cancer, *GC* gastric cancer, *ILD* interstitial lung disease, *UIP* usual interstitial pneumonia; ^a^ a case of HNC with possible UIP had grade 5 pneumonitis

Subsequently, we performed univariate and multivariate logistic regression analyses to evaluate risk factors for nivolumab-induced pneumonitis (Table 3). In the multivariate analysis, pre-existing ILD (odds ratio, 5.92; 95% confidence interval [CI], 2.07–18.54; *P* = 0.0008) and male sex (odds ratio, 5.58; 95% CI, 1.01–104.40; *P* = 0.049) were significant independent predictive factors for nivolumab-induced pneumonitis.

**Table 3:** Risk factors for nivolumab-induced pneumonitis in patients with solid tumors by univariate and multivariate logistic regression analysis

		Univariate model			Multivariate model		
		OR	(95% CI)	*P*-value	OR	(95% CI)	*P*-value
Age, years	≥65 vs. < 65	3.05	(1.08–10.90)	0.034	1.53	(0.44–6.11)	0.51
Sex	Male vs. female	10.13	(2.03–183.77)	0.0018	5.58	(1.01–104.40)	0.049
Smoking status	Current/ex-smoker vs. Never-smoker	1.52	(0.53–5.50)	0.45			
Performance status	≥2 vs. 0 or 1	0.75	(0.17–2.38)	0.64			
Tumor types	NSCLC vs. HNC/GC	1.79	(0.73–4.70)	0.21			
Pre-existing ILD	Yes vs. No	9.42	(3.67–25.80)	< 0.0001	5.92	(2.07–18.54)	0.0008
NLR^a^	≥2.5 vs. < 2.5	0.87	(0.35–2.22)	0.76			
	≥5.0 vs. < 5.0	0.68	(0.21–1.82)	0.45			
LDH (IU/l)	≥240 vs. < 240	1.52	(0.59–3.74)	0.37			
CRP (mg/dl)	≥1 vs. < 1	0.71	(0.27–1.75)	0.46			

*Sq* squamous cell carcinoma, *ILD* interstitial lung disease, *NLR* neutrophil-to-lymphocyte ratio, *LDH* lactate dehydrogenase, *CRP* C-reactive protein, *OR* odds ratio, *CI* confidence interval

^a^One patient with HNC was excluded because NLR was not performed

## Discussion

In this study, the overall incidence of all-grade pneumonitis was 11.7%, similar to a previous retrospective Japanese cohort study in patients with NSCLC treated by nivolumab [10, 11]. Although there was no significant difference in the univariate analysis, the NSCLC group tended to have a higher incidence of pneumonitis (14.6%) compared with the HNC/GC group (8.7%), which was related to the high trend of complicating pre-existing ILD in the NSCLC group (*P* = 0.047).

The present analysis revealed that pre-existing ILD and male sex were significant independent risk factors for nivolumab-induced pneumonitis in patients with solid tumors. Previous evidence has strongly indicated that pre-existing ILD is a risk factor for chemotherapy-induced pneumonitis, and these reports were particularly prevalent in lung cancer [12–17]. These findings are related to the fact that both lung cancer and ILD are closely associated with cigarette smoking. There is substantial epidemiological evidence that patients with ILD have a high risk of lung cancer [18, 19], whereas it has been reported that 10–20% of patients were complicated with ILD when lung cancer was diagnosed [13, 20]. Underpinning this epidemiological fact is that lung cancer and ILD share multiple common genetic, molecular, and cellular processes, such as epithelial-mesenchymal transition, endoplasmic reticulum stress, transforming growth factor expression, and oxidative stress [21, 22]. However, to our knowledge, there are few reports on the development of non-lung cancer and ILD. In our study, there was no significant difference in smoking rates between the NSCLC and HNC/GC groups, but the comorbidity rate of pre-existing ILD was significantly higher in the NSCLC group than in the HNC/GC group. The high prevalence of ILD in the NSCLC group, despite no difference in smoking history, may supports a correlation between the development of NSCLC and ILD. We previously reported that pre-existing ILD is a risk factor for anti-PD-1-induced pneumonitis [7]. However, it has not been elucidated whether pre-existing ILD is at risk of pneumonitis when using anti-PD-1 agents for other type of tumors. In previous phase III clinical trials, HNC and GC had a lower incidence of nivolumab-induced pneumonitis compared with NSCLC [2–5]. While a recent meta-analysis indicated that anti-PD-1-related pneumonitis of all grades develops more frequently in NSCLC and renal cell cancer than in melanoma [23, 24]. In our analysis based on pre-existing ILD on chest CT, NSCLC and HNC/GC showed similar trends in the development of pneumonitis. Interestingly, in patients with no fibrosis, the incidence of nivolumab-induced pneumonitis was approximately 5% in both NSCLC and HNC/GC, similar to results reported in previous large clinical trials. Moreover, in patients with pre-existing ILD, the incidence of nivolumab-induced pneumonitis jumped to approximately 30%. Although the biological mechanism of ICI-induced pneumonitis is poorly understood, it can be hypothesized that deregulation of immune effectors and T-cells in the lung interstitium is involved, leading to the increased production of inflammatory cytokines and resulting in an inflammatory response. Patients with ICI-induced pneumonitis were reported to have an increased number of activated T-cells in their bronchoalveolar lavage fluid compared with healthy control subjects, therefore T-cells are thought to participate in the onset of ICI-induced pneumonitis [25]. The activation of various immune cells likely targets the fragile lungs of chronic ILD patients, causing ICI-induced pneumonitis. The results of our study support the hypothesis that the risk of developing pneumonitis depends on the patient’s background lung condition, regardless of the tumor type.

It should be emphasized that increased risk of anti-PD-1-induced pneumonitis is not synonymous with the avoidance of anti-PD-1 treatment. Previous studies have demonstrated that the development of irAEs is associated with clinical benefit of ICIs in melanoma and NSCLC [26–31]. In a recent retrospective cohort study in patients with NSCLC treated with nivolumab (*n* = 613), landmark analyses of PFS at 2 months revealed no significant differences in PFS between patients with or without pneumonitis (7.9 vs. 5.9 months; *P* = 0.872). Moreover, in our previous report, patients with anti-PD-1-induced pneumonitis had relatively favorable survival outcomes [7]. Most cases of pneumonitis associated with nivolumab are relatively mild and classified as grade 1 to 2. It has been reported that nivolumab-induced pneumonitis responds well to steroid therapy and its mortality rate is low compared with gefitinib-induced pneumonitis, which is characterized by rapid progression and a high mortality rate of around 30 to 40% [12]. Therefore, ICI-induced pneumonitis may not lead to poor outcomes, unlike pneumonitis with a cytotoxic chemotherapy or molecularly targeted therapy that has been reported to have a poor prognosis [32]. Therefore, indications for ICI treatment should be judged comprehensively, taking into consideration pre-existing ILD as well as pulmonary function, which affects the severity of pneumonitis. All severe cases of pneumonitis (grade 3 or higher) were observed in patients with pre-existing ILD. Patients with pre-existing ILD were at increased risk of developing pneumonitis, and these patients also had impaired respiratory function, suggesting that pneumonitis was more likely to become severe.

A history of thoracic radiotherapy would also affect the incidence of chemotherapy-related pneumonitis in patients with NSCLC. Tamiya et al. reported that the incidence of nivolumab-induced pneumonitis was significantly higher in a group with thoracic radiation history than in a group without thoracic radiation history [10]. A higher incidence of nivolumab-induced pneumonitis in patients with thoracic radiation history may be because nivolumab can induce radiation recall pneumonitis. Therefore, our analysis excluded patients with a history of thoracic radiation to prevent bias.

Male sex has been reported as a risk factor for drug-induced pneumonitis in several studies following treatment with gefitinib, panitumumab, and pemetrexed [33–35]. However, we should keep in mind that males have a higher incidence of smoking than females, and multivariate analysis may not completely eliminate this bias. In the present study, only two out of 55 female patients (3.6%) had pre-existing ILD, and these two patients were classified as “inconsistent with UIP.” Therefore, to investigate the relationship between pre-existing ILD and nivolumab-induced pneumonitis particularly in females, further validation of another cohort is needed.

There are several limitations in the present study. First, this was a single-site retrospective study that used chart reviews of a relatively small patient cohort. Second, the number of patients with HNC/GC was smaller, since nivolumab was approved earlier for NSCLC than HNC/GC. NSCLC had a larger sample size and a higher incidence of pneumonitis than HNC and GC, which may have affected the overall results. In addition, the number of cases of HNC and GC is relatively small, and data on the incidence of pneumonitis in HNC/GC should be carefully interpreted. To compare the incidence of pneumonitis for each type of cancer, it is necessary to analyze it in a larger population. Third, we excluded patients who previously received thoracic radiation or other ICIs to prevent biases; however, since the standard treatment differs for each cancer type it is possible that the effects of prior treatment could not be completely excluded. Fourth, the patient cohort was across multiple cancer types, which prevented cancer-specific survival analysis. Fifth, lung function tests were not included in this analysis as some patients with HNC were unable to undertake them because of their post-tracheostomy status so they were not routinely performed in our institution. Sixth, bronchoalveolar lavage fluid was not routinely collected at our hospital and could not be incorporated into the analysis.

## Conclusions

Our findings showed that pre-existing ILD is a common risk factor for nivolumab-induced pneumonitis in solid cancer treatment, including NSCLC, HNC, and GC. The difference in the incidence of anti-PD-1-induced pneumonitis across various tumor types seems to have occurred due to the difference in the pre-existing ILD complication rate. To investigate the association between nivolumab-induced pneumonitis and pre-existing ILD across whole tumors, further analyses in a larger multicenter collaborative study are required. Likewise, further investigations are also required for anti-PD-L1-antibodies and chemo-ICI combination therapies to examine the relationship between pre-existing ILD and ICI-induced pneumonitis.

## Supplementary Information

**Additional file 1.**

## Data Availability

The datasets generated and/or analyzed during the current study are not publicly available due to a potential infringement of privacy but are available from the corresponding author on reasonable request.

## Abbreviations

ILD: Interstitial lung disease
NSCLC: Non-small cell lung cancer
PD-1: Programmed death 1
PD-L1: Programmed death ligand-1
HNC: Head and neck cancer
GC: Gastric cancer
ICI: Immune check point inhibitors
irAE: Immune-related adverse events
CT: Computed tomography
UIP: Usual interstitial pneumonia
COPD: Chronic obstructive pulmonary disease
CI: Confidence interval
