# Supplementary material for: Pre-existing interstitial lung disease is associated with onset of nivolumab-induced pneumonitis in patients with solid tumors: a retrospective analysis
Source: BMC Cancer. 2021 Aug 16;21:924. doi: 10.1186/s12885-021-08661-3 (PMC8369733; doi:10.1186/s12885-021-08661-3)
Supplement: Supplementary file 1 — Additional file 1. [file 12885_2021_8661_MOESM1_ESM.docx]

| **Supplemental Table S1. Patient characteristics of head and neck cancer and gastric cancer** | | |
| --- | --- | --- |
| Characteristics | HNC  n=43  n, (%) | GC  n=49  n, (%) |
| Age, years |  |  |
| Median (range) | 64 (24–84) | 68 (33–85) |
| <65 | 23 (53.5) | 15 (30.6) |
| ≥65 | 20 (46.5) | 34 (69.4) |
| Sex |  |  |
| Male | 33 (76.7) | 32 (65.3) |
| Female | 10 (23.3) | 17 (34.7) |
| Smoking status |  |  |
| Current/ex-smoker | 36 (83.7) | 31 (63.3) |
| Current smoker | 5 (11.6) | 6 (12.2) |
| Ex-smoker | 31 (72.1) | 25 (51.0) |
| <20 pack-years | 10 (23.3) | 8 (16.3) |
| ≥20 pack-years | 24 (55.8) | 21 (42.9) |
| Unknown pack-years | 2 (4.7) | 2 (4.1) |
| Never-smoker | 7 (16.3) | 18 (36.7) |
| Performance status |  |  |
| 0–1 | 34 (79.1) | 40 (81.6) |
| ≥2 | 9 (20.9) | 9 (18.4) |
| Prevalence of patient comorbidities |  |  |
| Arterial hypertension | 11 (25.6) | 15 (30.6) |
| Cardiovascular disease | 4 (9.3) | 4 (8.2) |
| Diabetes | 7 (16.3) | 5 (10.2) |
| COPD | 3 (7.0) | 0 |
| No. of treatment cycles of nivolumab |  |  |
| Median (range) | 6 (1–25) | 4 (1–23) |
| Pre-existing ILD on chest CT |  |  |
| Normal | 35 (81.4) | 43 (87.8) |
| Pre-existing ILD | 8 (18.6) | 6 (12.2) |
| UIP | 1 (2.3) | 1 (2.0) |
| Possible UIP | 1 (2.3) | 0 |
| Inconsistent with UIP | 6 (14.0) | 5 (10.2) |
| HNC, head and neck cancer; GC, gastric cancer; COPD, chronic obstructive pulmonary disease; ILD, interstitial lung disease; CT, computed tomography; UIP, usual interstitial pneumonia | | |
